# The efficacy and safety of Chinese herbal medicine as an add-on therapy for amyotrophic lateral sclerosis: An updated systematic review and meta-analysis of randomized controlled trials

**DOI:** 10.3389/fneur.2022.988034

**Published:** 2022-10-06

**Authors:** Yingdi Liao, Sijin He, Duo Liu, Lihua Gu, Qigang Chen, Shuang Yang, Daiying Li

**Affiliations:** Department of Rehabilitation, Kunming Municipal Hospital of Traditional Chinese Medicine, Kunming, China

**Keywords:** Chinese herbal medicine, amyotrophic lateral sclerosis, meta-analysis, systematic review, randomized controlled (clinical) trial

## Abstract

**Background:**

Amyotrophic lateral sclerosis (ALS) has attracted widespread attention because of its unknown pathogenesis, rapid progression, and life-threatening and incurable characteristics. A series of complementary therapies, including Chinese herbal medicine (CHM), is available for use in the clinic and has been the focus of much research. However, it is unclear as to whether supplementary CHM relieves disease symptoms or extends life span; thus, we conducted this updated meta-analysis to validate the efficacy and safety of this practice.

**Methods:**

We searched six electronic databases for randomized controlled trials involving CHM and patients with ALS that were published up to April 2022. Two researchers independently screened the literature, assessed the risk of bias for each trial, and then extracted data. The methodological quality of the included trials was assessed using the Cochrane risk of bias tool, and a pooled data analysis was performed using RevMan 5.3.

**Results:**

A total of 14 trials led to the publication of 15 articles featuring 1,141 participants during the study period; the articles were included in the systematic review. In terms of increasing ALS functional rating scale (ALSFRS) scores, CHM was superior to the placebo after 3 months of treatment [mean difference (MD):0.7; 95% CI:0.43 to 0.98; *P* < 0.01] and to riluzole after 4 weeks of treatment (MD: 2.87; 95% CI: 0.81 to 4.93; *P* < 0.05), and it was superior to conventional medicine (CM) alone when used as an add-on therapy after 8 weeks of treatment (MD: 3.5; 95% CI: 0.51 to 6.49; *P* < 0.05). The change in the modified Norris score (m-Norris) from baseline to the end of more than 3 months of treatment was significantly different when compared between the CHM plus CM group and the CM alone group (MD: 2.09; 95% CI: 0.62 to 3.55; *P* < 0.01). In addition, CHM had a significantly better effect on increase in clinical effective rate (RR: 1.54; 95% CI: 1.23 to 1.92; *P* < 0.01) and improvement in forced vital capacity (MD: 7.26; 95% CI: 2.92 to 11.6; *P* < 0.01). However, there was no significant difference between the CHM therapy and CM in terms of improving life quality (MD: 5.13; 95% CI: −7.04 to 17.31; *P* = 0.41) and decreasing mortality (RR: 0.41; 95% CI: 0.04 to 4.21; *P* = 0.46).

**Conclusion:**

The analysis suggested that the short-term adjunct use of CHM could improve the ALSFRS score and clinical effect with a good safety profile when compared with the placebo or riluzole alone. However, future research should be centered on the long-term efficacy of patient-oriented outcomes.

**Systematic review registration:**

https://www.crd.york.ac.uk/PROSPERO/display_record.php?RecordID=323047, identifier: CRD42022323047.

## Introduction

Amyotrophic lateral sclerosis (ALS) is a progressive neurodegenerative condition involving upper motor neurons (UMNs) and lower motor neurons (LMNs) in the cerebral cortex, brain stem, and spinal cortex. The clinical neurological manifestations of ALS include muscle stiffness, spasm, hyperreflexia, and pathological reflexes as a sign of UMN injury and muscle weakness and atrophy as a sign of LMN damage with difficulty walking, speaking, and swallowing (1, 2). The prognosis of ALS is fatal, resulting in death due to respiratory failure or other associated conditions. Furthermore, there is no cure for ALS (1). This condition progresses rapidly, and the median survival duration is around 3–5 years from primary symptoms to death (3). This disease has an incidence of 2.5 per 100,000 persons-years and is more prevalent in men than in women (4). The cause of ALS has yet to be elucidated; however, ~5–10% of patients with ALS are thought to suffer from a familial form of ALS that exerts familial genetic characteristics and feature mutations in several genes including *C9orf72, SOD1, FUS*, and *TARDBP*. The remaining 90% of patients are considered as sporadic cases of ALS associated with abnormalities of the immune system, toxic exposure, mitochondrial dysfunction, oxidative stress, neuroinflammation, or glutamate poisoning (2–5).

Riluzole, an anti-glutamate drug, is currently the only drug approved by the Food and Drug Administration (FDA) to prolong the life span of patients with ALS. However, this drug is expensive and is associated with several detrimental side effects including fatigue and diarrhea (4). Furthermore, an increasing body of evidence supports the fact that antioxidants may represent a potential treatment without serious clinical adverse effects although the efficacy of this treatment remains uncertain (6). Consequently, there is an urgent need to identify new and effective integrated therapies to slow the progression of ALS and increase the survival rate of patients. According to a previous survey on the use of integrated therapies conducted in Shanghai, China, Traditional Chinese Medicine (TCM) is the most commonly used form of complementary and supplementary therapy for treating ALS (7).

The clinical symptoms of ALS suggest that it could be defined as a “wilt disease” in the TCM system. Chinese Herbal Medicine (CHM), a form of TCM therapy, has been used to treat a wilt disease for thousands of years (8). A recent scoping review analyzed the research trends for the use of traditional herbal medicine in the treatment of ALS and identified many case reports, clinical observation studies, and randomized controlled trials (RCTs) involving the use of traditional herbal medicine for the treatment of ALS in East Asia, thus indicating that CHM may represent a potentially unique and effective therapy for ALS (4). However, the mechanisms and targets of CHM in the treatment of ALS were not described in this previous scoping review. A preclinical study showed that TCM compounds could inhibit the deleterious aggregation of abnormally phosphorylated nerve filaments around the nucleus, thus maintaining the integrity of the cytoskeleton, reducing axonal atrophy, and improving axoplasmic transport to delay neuronal degeneration (9). Over recent years, single TCM drugs, Chinese medicine compounds, Chinese patent medicines, and injections of Chinese medicines have all been proven to be effective in the treatment of ALS in clinical trials. However, there is insufficient evidence to support the efficacy and safety of CHM for ALS. Thus, it is necessary to evaluate the documented efficacy of this form of treatment by performing a systematic review and meta-analysis of RCTs. We aimed to answer the following questions: (1) whether CHM can improve clinical symptoms in patients with ALS, (2) whether CHM reduces mortality in patients with ALS, (3) whether CHM improves the quality of life of patients with ALS, (4) whether CHM improves the clinical effective rate of patients with ALS, and (5) whether CHM is safe when used alone or as an adjunct therapy.

## Methods

The review protocol was registered on PROSPERO (Reference: CRD42022323047) and was performed according to the checklist of Preferred Reporting Items for Systematic Reviews and Meta-Analyses (10).

### Eligibility criteria

#### Types of study

Randomized controlled trials (RCTs) on Chinese herbal medicine for ALS; the publication language was restricted to English and Chinese.

#### Participants

Participants with ALS of any age and race were eligible. The included trials needed to provide definite and universal diagnostic criteria for ALS formulated by the World Federation of Neurology Sub-Committee on Neuromuscular Diseases at El Escorial (11) or the Neurology Branch of the Chinese Medical Association (12).

#### Interventions

The Chinese herbal medicine was used on the treatment group alone or in combination with a conventional medicine (CM) such as riluzole, mecobalamin, vitamin, and other antioxidants. The Chinese herbal medicine included Chinese patent medicine, traditional herbal decoction, and other single Chinese medicines or compounds. The route of administration for the Chinese herbal medicine was not restricted. The controls included placebo or routine pharmacotherapies such as riluzole, mecobalamin, vitamin, and other antioxidants. When the treatment group was CHM plus another treatment, the adjunct therapy needed to be the same as the control.

#### Outcomes

The primary outcome was the assessment of functional changes in patients with ALS, as appraised with the ALS functional rating scales (ALSFRS) or the revised ALS functional rating scales (ALSFRS-r) and modified Norris scale (m-Norris) at the end of the treatment duration or after the follow-up. The ALSFR is a 10-item instrument, while the ALSFRS-r is a 12-item instrument that integrates all aspects of myelination symptoms, motor symptoms, and respiratory symptoms. The ALSFRS scores range from 0 to 40 (ALSFRS-r from 0 to 48); higher scores indicate less severe neurological impairment (13). m-Norris is a comprehensive scale that is used mainly to evaluate myelination and limb function; the score ranges from 0 to 99, with lower values implying more severe functional impairment (14).

The secondary outcomes were as follows: (1) the overall clinical effective rate was defined according to universally approved criteria, which are divided into markedly effective, effective, and ineffective; (2) changes in the number of points allocated for TCM syndrome as assessed by main and minor symptoms. The main symptoms include four items: fleshy atrophy, wilting limb, swallowing difficulty, and sluggish speech; these are graded as 0, 2, 4, and 6 points, respectively. The minor symptoms include shortness of breath, fatigue, spontaneous sweating, thirst, dry throat, being upset, waist knee soreness, and tinnitus; each item is graded as 0, 1, 2, and 3 points; (3) the assessment of life quality in patients with ALS; (4) pulmonary function, as assessed by forced vital capacity (FVC); (5) composite endpoint events including mortality, and noninvasive or invasive ventilation; (6) adverse events (AEs).

### Exclusion criteria

Articles were excluded for the following reasons: (1) repeated publications and not related to ALS, (2) involved animal research or were not RCTs, (3) devoid of relevant outcomes or provided an incomplete dataset for analysis, and (4) the treatment group included acupuncture, moxibustion, massage, or treatments other than traditional Chinese medicine.

### Study selection and data extraction

A systematic and comprehensive literature search was performed from inception to March 2022 using a range of online databases including PubMed, Embase, the Cochrane Library, China National Knowledge Infrastructure, Wangfang, and the China Biomedical Database. The search terms were as follows: amyotrophic lateral sclerosis, Chinese herbal, preparation, and randomized controlled trials. The retrieval strategy is shown in Appendix. Additional manual searches were based on previous related studies (4). Two authors (YL and SH) identified and selected the studies. Based on the above inclusion and exclusion criteria, titles and abstracts were identified and checked by the two authors. The full text of all relevant articles was obtained and screed by the reviewers. Disagreements were resolved by discussion.

Data extraction was performed independently and involved a cross-check by the two authors (YL and SH). Then, we extracted the following information from eligible studies into Microsoft Excel 2007: (1) basic information including authors, publication year, study design, and diagnostic criteria of ALS; (2) patient characteristics including total number, age, gender, and disease history; (3) types of interventions including intervention name, dose, treatment duration, frequency, usage, and follow-up; (4) outcomes and AEs. Disagreements were resolved by discussion.

### Risk of bias assessment

The two authors (YL and SH) assessed the risk of bias in the included studies by applying tools developed by the Cochrane Collaboration. These tools covered the following seven domains: random sequence generation, allocation concealment, blinding of participants and personnel, blinding of outcome assessment, incomplete outcome data, selective reporting, and other forms of bias. The risk of bias judgments was categorized as low, high or, unclear. Disagreements were resolved by discussion.

### Statistical analysis

Data analysis was performed with the Review Manager software (RevMan version 5.3; The Cochrane Collaboration, Oxford, United Kingdom). Continuous data are presented as the mean difference (MD) with 95% CI, while dichotomous data are presented as relative risk (RR) with 95% CI. The heterogeneity of the included RCTs was evaluated by chi-squared tests. Fixed-effects models were used to estimate the effect for studies with low heterogeneity (I^2^ < 50%, *P* > 0.1). If the I^2^ value was more than 50%, we performed a subgroup analysis to identify the source of the heterogeneity. In addition, a subgroup analysis was carried out for different durations, and random-effects models were used for estimation. A sensitivity analysis was performed to assess the robustness of results by only including trials with a low risk of bias for blinding of participants and allocation concealment. Funnel plots were used to assess publication bias if more than 10 RCTs reported the same outcome.

## Results

### Literature search

The initial search identified 3,586 articles from the selected databases. According to the eligibility criteria, a total of 26 full texts were further screened after checking the titles and abstracts. Twelve articles were excluded for the following reasons: not RCTs, contained invalid data, featured duplicate data, reported unrelated outcomes, and inappropriate controls. In some cases, the full text was not available. Finally, 15 articles were included in our analysis (15–29). However, two articles (17, 18) published different outcomes for the same trial. Therefore, a total of 14 original studies were included in our meta-analysis. Supplementary Figure 1 shows a flowchart that describes the strategy used for screening articles.

### Characteristics of the included studies

All of the included studies, featuring 1,141 participants, were RCTs and were published between 2006 and 2020 (15–29). One study was published in English (28), while the remaining articles were published in Chinese. The treatment duration ranged from 4 weeks to 6 months. Ten studies reported follow-up data at the end of treatment (16, 20–28). Nine articles mentioned the course of ALS within 5 years (15–19, 22, 24, 27, 29). In three trials, the treatment group was treated with CHM combined with riluzole (16, 21, 22). One trial combined CHM with a coenzyme, vitamin E, and mecobalamine (20). Eight trials used only CHM in the experimental group (15, 17–19, 24–26, 28, 29). Two trials were double-blinded, double-dummy RCTs (23, 27). The control group was treated with conventional medicine alone or with a CHM placebo. The most frequent herb was *huangq*i, followed by *baizhu, shengdi, rensheng, yingyanghuo, dansghe*, and *fuling*. The most frequent Chinese preparations were Jia Wei Si Jun Zi decoctions, Ji Wei Ling preparations, and Fu Yuan Sheng Ji granules. The characteristics of the included studies are shown in Tables 1, 2.

**Table 1:** Basic features of the included studies.

**Included studies**	**Sample size (T/C)**	**Ages: mean (SD)/range, T/C**	**Gender (M/F)**		**Course of disease (month)**	**Followup (day)**	**Outcome**	
			**T**	**C**			**Primary outcome**	**Secondary outcome**
Zhang (15)	42/42	45.36(6.52)/45.51(6.38)	34/8	36/6	6–60	NA	ALSFRS-r, clinical effect	
Li et al. (16)	30/28	56.43(11.08)/55.79(10.06)	19/11	19/9	2–36	540	m-Norris,	FVC, Endpoint events
Wang et al. (17)	100/25	55.11(13.52)/56.64(11.18)	60/40	16/9	< 60	NA	Clinical effect	TCM syndrome score, AEs
Ren et al. (18)	100/25	55.11(13.52)/56.64(11.18)	60/40	16/9	< 60	NA	m-Norris, Clinical effect	AEs
Chen et al. (19)	240/80	54.67(10.23)/53.56(10.31)	147/93	46/34	6–36	NA	Clinical effect	
Zhu et al. (20)	24/21	NA	15/9	14/7	NA	365	ALSFRS-r	AEs
Wang et al. (21)	30/30	53.07(11.19)/53.07(11.19)	32/28		NA	56	ALSFRS-r	Endpoint events, AEs,FVC
Su et al. (22)	25/10	60.2(14.1)/59.4(9.0)	13/12	6/4	< 60	90	m-Norris	TCM syndrome score
Pan (23)	38/38	49.38(8.96)/50.05(8.10)	26/12	25/13	NA	90	ALSFRS, m-Norris, Clinical effect	Life quality, AEs
Wang (24)	30/30	44.57(9.55)/48.13(8.54)	20/10	22/8	3–60	28	ALSFRS, Clinical effect	Life quality, FVC, AEs
Sui et al. (25)	33/31	54.00(11.96)/54.00(11.92)	18/15	20/11	NA	84	m-Norris, Clinical effect	TCM syndrome score, AEs
Wu et al. (26)	16/16	41.1/42.9	13/3	12/4	NA	90	Clinical effect	
Ma (27)	30/30	48.33(10.24)/48.68(11.06)	9/21	10/20	5–28	28	ALSFRS, m-Norris, Clinical effect	Life quality, FVC, AEs
Pan et al. (28)	23/19	51.6(7.2)/50.1(4.2)	14/9	11/8	NA	180	ALSFRS-r	Life quality, Endpoint events, AEs
Wenjie et al. (29)	40/40	24~76/20~79	25/15	27/13	< 60	NA	m-Norris, Clinical effect	TCM syndrome score,

T, treatment group; C, control group; NA, data are not available; M, male; F, female; ALSFRS-r, revised ALS functional rating scales; ALSFRS, ALS functional rating scales; m-Norris, modified Norris scales; FVC, forced vital capacity; AEs, adverse events; TCM, Traditional Chinese Medicine.

**Table 2:** Details of interventions in the included studies.

**Included studies**	**Interventions**		**Principle of syndrome differentiation and treatment**	**Ingredients of the treatment group**	**Treatment duration**
	**T**	**C**			
Zhang (15)	Shen Mai injection 20 ml ivd qd + neurotrophin 2000 u ivd qd+ Cobamamide 0.5mg po qd + adenosine triphosphate disodium tablets 40 mg po qd	Neurotrophin 2000 u ivd qd+ Cobamamide 0.5mg po qd + adenosine triphosphate disodium tablets 40 mg po qd	NA	*hongsheng, maidong*	45 d
Li et al. (16)	Fu Yuan Sheng Ji granules 1 pack po bid + Riluzole tablets 50 mg po bid	Riluzole tablets 50 mg po bid	Fortify spleen and tonify kidney	*huangqi, yingyanghuo, baizhu, shanyurou, shengdi, xianlingpi, gancao*	180 d
Wang et al. (17)	Fu Yuan Sheng Ji granules 15 g po bid	Riluzole tablets 50 mg po bid	Fortify spleen and tonify kidney	*huangqi, yingyanghuo, baizhu, shanyurou, shengdi, xianlingpi, gancao*	84 d
Ren et al. (18)	Fu Yuan Sheng Ji granules 15 g po bid	Riluzole tablets 50 mg po bid	Fortify spleen and tonify kidney	*huangqi, yingyanghuo, baizhu, shanyurou, shengdi, xianlingpi, gancao*	84 d
Chen et al. (19)	Ji Wei Ling injection 48 ml qd ivd+ Ji Wei Ling capsule	Riluzole tablets 50 mg po bid + neurotrophin 2000 u ivd qd	Reinforce source qi, tonify nutrient aspect and promote muscle vitality	*rensheng, lurong, heshouwu*	75 d
Zhu et al. (20)	Jia Wei Si Jun Zi decoction 1 pack + coenzyme Q10 + vitamin E + mecobalamine	Placebo + coenzyme Q10 + vitamin E + mecobalamine	Fortify spleen and tonify qi	*huangqi, dangshen, baizhu, fuling, zhigancao, danggui*	9 m
Wang et al. (21)	Jian Pi Yi Fei granules 1 pack po bid + Riluzole tablets 50 mg po bid	Riluzole tablets 50 mg po bid	Fortify spleen and tonify lung	*huangqi, dangshen, baizhu, wuweizi, duzhong, tusizi, maidong, chenpi, fabanxia, baijiangcan, xinren, jiegeng, chaihu, zhimaqianzi, zhigancao*	8 w
Su et al. (22)	Yi Qi Qiang Ji decoction 1 pack po bid + Riluzole tablets50 mg po bid	Riluzole tablets 50 mg po bid	Tonify qi and nourish ying and yang	*huangqi, dangshen, fuling, gancao, shengdi, yingyanghuo, zhidahuang, shengma*,	90 d
Pan (23)	Shen Zhe Jiang Qi powder 5 g po tid +Riluzole placebo 50mg po tid	Shen Zhe Jiang Qi placebo 5 g po tid + Riluzole tablets 50mg po tid	Regulate qi	*dangshen, zheshi, guiban, roucongrong, shanzhuyu, roucongrong, jiegeng, zhike, wugong*	90 d
Wang (24)	Ji Wei Ling injection 24 ml ivd qd	Riluzole Tablets 50 mg po bid	Reinforce source qi, tonify nutrient aspect and promote muscle vitality	*rensheng, lurong, heshouwu*	28 d
Sui et al. (25)	Huo Ling Sheng Ji decoction 1 pack po bid	Riluzole tablets 50 mg po bid	Replenish qi and tonify spleen, warm kidney	*huangqi, yingyanghuo, fuchaobaizhu, shanzhuyu, fuling, shengdihuang*	12 w
Wu et al. (26)	Huangqi powder 60 g po tid	Riluzole Tablets 50 mg po bid	Tonify spleen, lung and kidney, replenish qi and blood	*huangqi*	90 d
Ma et al. (27)	Ji Wei Ling injection 32 ml ivd qd + Riluzole placebo 50 mg po bid	Ji Wei Ling injection placebo 32 ml ivd qd+Riluzole tablets 50 mg po bid	Reinforce source qi, tonify nutrient aspect and promote muscle vitality	*rensheng, lurong,heshouwu*	28 d
Pan et al. (28)	Jia Wei Si Jun Zi decoction 1 pack po bid	Riluzole tablets 50 mg po bid	Nourish spleen and enrich vitality	*huangqi,renshen, baizhu, fuling, zhigancao, roucongrong*	6 m
Wenjie et al. (29)	Zi Ni Fang 1 pack po bid	Riluzole tablets 50 mg po bid	Tonify kidney, fortify the spleen and soothe the liver	*huangqi, shengdihuang, xianlingpi, bajitian, shanzhuyu, fuling, shihu, huainiuxi, chaihu, yujin*	6m

T, treatment group; C: control group; NA, data are not available; d, days; m, months; w, weeks.

### Risk of bias assessment for the included studies

Eleven studies (15–18, 20, 21, 23–25, 27–29) reported appropriate methods of random sequence generation, including random number tables and computer software. Three other studies (19, 22, 26) mentioned randomized trials but did not report the randomization methods in detail. Allocation concealment was unclear in all the studies because of limited information, although one study (23) reported the use of sequentially numbered containers to conceal allocation. Three trials (15, 19, 24) reported a high risk of blinding because blindness could have been disrupted by applying different methods of administration in the control and treatment groups. Three trials (20, 23, 27) were reported in a double blinded manner to participants and personnel; we considered this as a low risk. However, the remaining studies (15–19, 21, 22, 24–26, 28, 29) did not report blinding to the participants and personnel. One trial (27) reported the blinding of outcome assessment, while the other 10 trials (16–18, 20–23, 25, 26, 28, 29) lacked detailed information related to the blinding of outcome assessment; we considered this as an unclear risk. One trial (20) reported a dropout but did not provide a clear reason for the dropout. The reporting bias for all trials (15–29) was assessed as a low risk because they all reported predefined outcomes in their Methods sections. For all the studies, other forms of bias were considered to be an unclear risk because of lack of information. Detailed results related to risk of bias are shown in Supplementary Figures 2, 3.

### Effects of interventions

#### Primary outcome measures

##### CHM alone vs. controls

**Figure 1:**
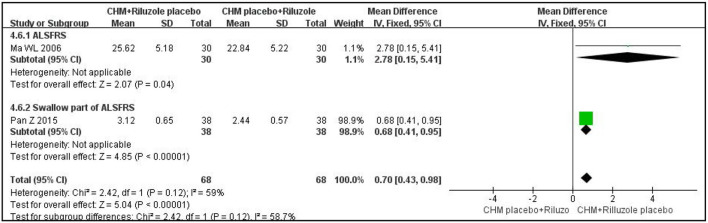
Forest plot of ALSRF for ALS compared CHM with controls. ALSFRS, ALS functional rating scales; CHM, Chinese herbal medicine; ALS, amyotrophic lateral sclerosis.

In terms of assessment of ALS function, one double-blind, double-dummy RCT with 60 participants measured outcome with the ALSRF scale and reported that CHM plus a placebo for riluzole was more effective than riluzole plus a placebo for CHM after 3 months of treatment (MD: 2.78; 95% CI: 0.15 to 5.41; *P* < 0.05) (27). Another RCT, with the same methodological design, measured the outcome with the swallowing item in ALSFRS; the results showed that the experimental group had a better effect on improving swallow function than the control group after 3 months of treatment (MD:0.68; 95% CI: 0.41 to 0.95; *P* < 0.01) (23). The results are shown in Figure 1. Another RCT with 60 participants showed that CHM was superior to riluzole in improving ALSRF scores after 4 weeks of treatment (MD: 2.87; 95% CI: 0.81 to 4.93; *P* < 0.05) (24). In terms of ALSRF-r scores, one trial with 48 participants showed that there was no significant difference between the CHM group and the riluzole group after 6 months of treatment (MD: 1.8; 95% CI: −2.62 to 6.22; *P* > 0.05) (28).

**Figure 2:**
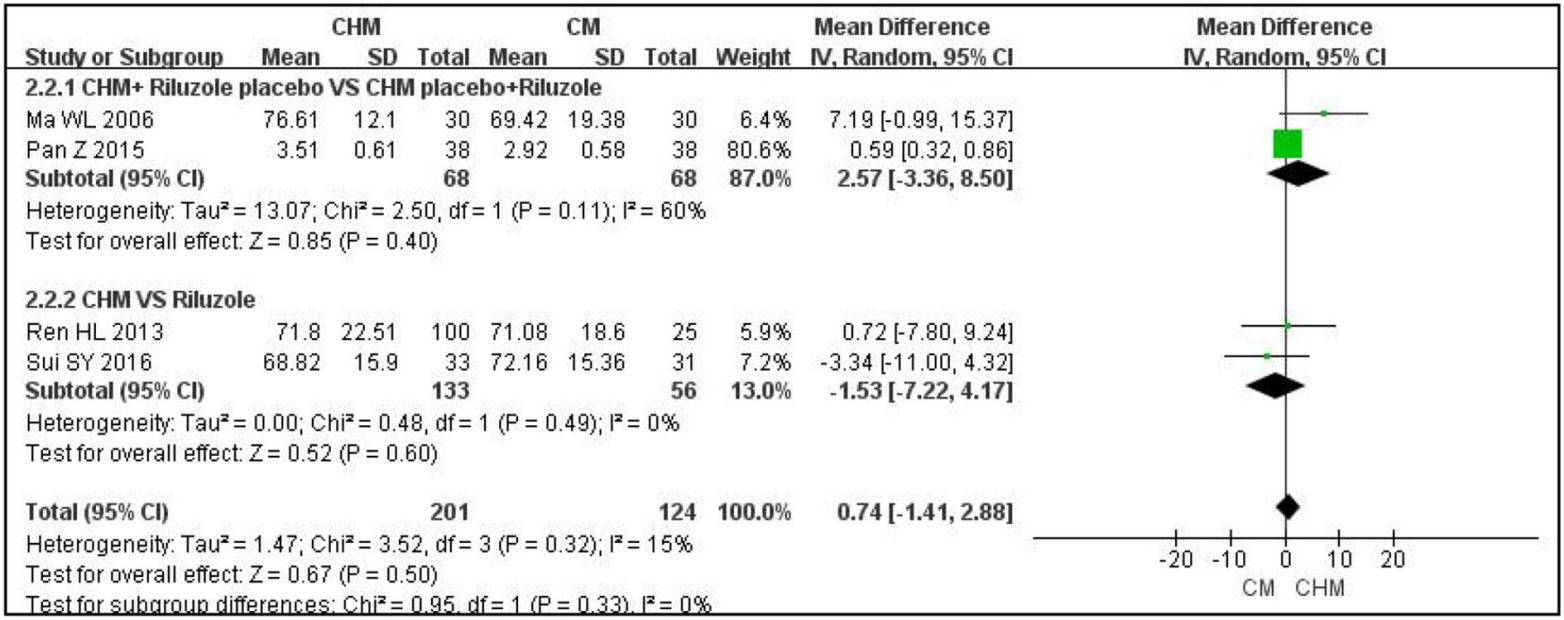
Forest plot of m-Norris for ALS compared CHM with controls. m-Norris, modified Norris scales; CHM, Chinese herbal medicine; ALS, amyotrophic lateral sclerosis.

With regard to ALS function, as measured with the m-Norris scale, CHM plus a placebo for riluzole was not superior to riluzole plus a placebo for CHM after 3 months of treatment (MD: 2.57; 95% CI: −3.36 to 8.5; *P* = 0.4; I^2^ = 60%; *n* = 2 RCTs; 136 participants) (23, 27). CHM alone was not better than riluzole in improving the m-Norris scale score after 12 weeks of treatment (MD: −1.53; 95% CI: −7.22 to 4.17; *P* =0.6; I^2^ = 0%; *n* = 2 RCTs; 189 participants) (18, 25). The results are shown in Figure 2. The other trial was not analyzed because the data were reported as medians rather than means; however, there was no significant difference between the CHM group and the riluzole group after 6 months of treatment (*P* > 0.05) (29).

##### CHM plus conventional medicine vs. controls

**Figure 3:**
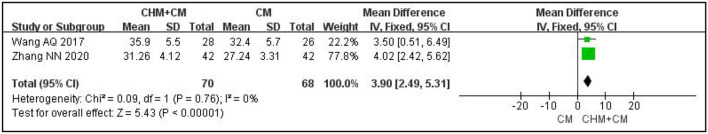
Forest plot of ALSFRS-r for ALS compared CHM plus CM with CM. r-ALSFRS, revised ALS functional rating scales; CHM, Chinese herbal medicine; ALS, amyotrophic lateral sclerosis; CM, conventional medicine.

With regard to ALS function, as assessed with the ALSFRS-r scale, one RCT featuring 45 participants showed that CHM was no more effective than a placebo based on CM after 9 months of treatment (MD: 5; 95% CI: −0.08 to 10.08; *P* = 0.05) and after 3 months of follow-up (MD: 1.24; 95% CI: −4.44 to 6.92; *P* = 0.67) (20). At the end of 7 to 8 weeks of treatment, CHM plus CM had a better effect than CM alone (MD: 3.9; 95% CI: 2.49 to 5.31; *P* < 0.001; I^2^ = 0%; *n* = 2 RCTs; 138 participants) (15, 21). The results are shown in Figure 3.

**Figure 4:**
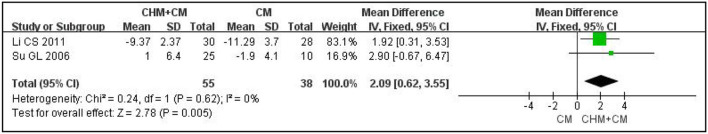
Forest plot of m-Norris for ALS compared CHM plus CM with CM. m-Norris, modified Norris scales; CHM, Chinese herbal medicine; ALS, amyotrophic lateral sclerosis; CM, conventional medicine.

With regard to ALS function, as assessed with the m-Norris scale, two trials reported changes from baseline to the end of treatment; adding CHM significantly increased this trend for change when compared to riluzole alone after 3 months of treatment (MD: 2.09; 95% CI: 0.62 to 3.55; *P* < 0.01; I^2^ = 0%; *n* = 2 RCTs; 93 participants) (16, 22). The results are shown in Figure 4.

#### Secondary outcomes

##### Overall clinical effective rate

###### CHM alone vs. controls

Two RCTs reported clinical effective rate as assessed by ALS symptoms and life quality; the clinical effect of CHM plus a placebo for riluzole was better than that of riluzole plus a placebo for CHM after 4 weeks of treatment (RR: 1.54; 95% CI: 1.23 to 1.92; *P* < 0.001; I^2^ = 0%; *n* = 2 RCTs; 136 participants) (23, 27). The results are shown in Supplementary Figure 4.

Six trials compared CHM with conventional therapy and assessed the overall effective rate with different criteria (17–19, 24–26, 29); four trials measured the effective rate with the m-Norris scale and showed that there was no significant difference between the two groups (RR:0.94; 95% CI: 0.6 to 1.47; *P* = 0.78; I^2^ = 16%; *n* = 4 RCTs; 301 participants) (18, 25, 26, 29). The remaining RCTs measured the effective rate with other criteria and showed that the CHM group had a better effect than the TCM group (as assessed by TCM syndrome points: RR: 2.75; 95% CI: 1.65 to 4.57; *P* < 0.001; I^2^ = 41%; *n* = 2 RCTs; 269 participants; as assessed by ALS clinical symptoms: RR: 22.78; 95% CI: 7.5 to 69.21; *P* < 0.01; *n* = 1 RCT; 320 participants; as assessed by ALS bulbar paralysis clinical symptoms scores: RR: 2.38; 95% CI: 1.24 to 4.56; *P* < 0.01; *n* = 1 RCT; 60 participants) (17, 19, 24, 25). The results are shown in Supplementary Figure 5.

###### CHM plus conventional medicine vs. controls

Only one trial reported the effective rate as assessed by ALS clinical symptoms and showed that the CHM plus CM group had a better effect than the CM group (RR: 1.26; 95% CI: 1.03 to 1.53; *P* < 0.05; 84 participants) (15).

###### Traditional Chinese medicine syndrome

One trial featuring 35 participants reported a change in TCM syndrome score from baseline to the end of treatment and showed that CHM plus riluzole had a better effect on reducing the change in TCM syndrome score than riluzole alone after 3 months of treatment (MD: 3.9; 95% CI: 1.77 to 6.03; *P* < 0.001) (22). Another trial showed that CHM had a better effect on reducing changes in TCM syndrome scores than riluzole after 6 months of treatment (*P* < 0.05) (29). However, we did not perform an analysis for this study because data were reported as medians rather than means (29). Two trials reported TCM syndrome score after 12 weeks of treatment; the pooled data showed that the effect of CHM on reducing the TCM syndrome score was better than that of riluzole (MD: −3.27; 95% CI: −5.4 to −1.14; *P* < 0.01; I^2^ = 0%; *n* = 2 RCTs; 189 participants) (17, 28). However, there was no significant difference between the two groups with regard to reducing fleshy atrophy symptom score and limb wilt score (fleshy atrophy symptom score: MD: −0.25; 95% CI: −0.73 to 0.24; *P* = 0.89; I^2^ = 0%; *n* = 2 RCTs; 189 participants; limb wilt symptom score: MD:0.19; 95% CI: −0.24 to.62; *P* = 0.38; I^2^ = 0%; *n* = 2 RCTs; 189 participants) (17, 28). The results are shown in Supplementary Figure 6.

###### Assessment of life quality of ALS patients

Two double-blinded, double-dummy trials reported the quality of life as measured with the ALS assessment questionnaire (ALSAQ-40) and showed that there was no significant difference between the experimental group and the control group (MD: 5.13; 95% CI: −7.04 to 17.31; *P* =0.41; I^2^ = 73%; *n* = 2 RCTs; 136 participants) (23, 27). The results are shown in Supplementary Figure 7. The other two trials compared CHM with riluzole but used different scales to measure life quality (24, 28); therefore, we did not perform a pooled analysis. However, the published results showed that there was a significant difference between the two groups [ALSAQ-40: MD: 3.33; 95% CI: −4.02 to 10.68; P = 0.37; *n* = 1 RCT; 60 participants; SF-36 physical functioning (PF) subscale: MD: 1.30; 95% CI: −2.7 to 5.3; *P* = 0.52; *n* = 1 RCT; 42 participants].

###### Pulmonary function as assessed by forced vital capacity

One trial featuring 60 participants reported FVC data and showed that CHM plus a placebo for riluzole improved the FVC in a manner more superior to that of riluzole plus a placebo for CHM after 4 weeks of treatment (MD: 7.26; 95% CI: 2.92 to 11.6; *P* < 0.01) (27). Wang et al. compared CHM plus conventional therapy with conventional therapy alone and showed that there was no significant differences between the CHM plus CM group and the CM alone group after 8 weeks of treatment (MD: 2.14; 95% CI: −7.93 to 12.21; *P* = 0.68) (21). Two trials reported FVC changes from baseline to the end of treatment (16, 24). Wang et al. reported that CHM had a better effect than riluzole on improving FVC values after 4 weeks of treatment (MD: 2.31; 95% CI:0.62 to 4; *P* < 0.01) (24). However, Li et al. showed that there were no significant differences between a CHM plus CM group and a CM alone group after 6 months of treatment (MD:0.84; 95% CI: −0.4 to 2.08; *P* = 0.18) (16).

###### Composite endpoint events

Three trials (16, 21, 28) reported the outcome after long-term treatment or follow-up including death, tracheotomy, gastrostomy, and coma. The incidence of non-favorable clinical events was 13.6%. One trial featuring 42 participants compared CHM alone with riluzole with regard to mortality and revealed that there was no significant difference between the two groups after 6 months of treatment (RR:0.41; 95% CI: 0.04 to 4.21; *P* = 0.46) (28). Another trial featuring 58 participants reported mortality due to respiratory failure at the end of 18 months follow-up and showed that there was no significant difference between the CHM plus riluzole group and the riluzole alone group (MD:0.35; 95% CI: 0.1 to 1.19; *P* = 0.09) (16). This trial also reported that three patients underwent tracheotomy in the experimental group (16). Wang et al. reported that two patients underwent tracheotomy with mechanical breathing, one underwent gastrostomy, and one patient who fell into a coma after injury, at the end of 2 months of follow-up but did not mention whether the event occurred in the control group or the treatment group (21).

###### Adverse events

Adverse events were monitored in eight studies (17, 18, 20, 21, 23–25, 27, 28). Two of these studies found no adverse events in the two groups (17, 18, 20). Five studies reported that the incidence of adverse events in the CHM group was around 2%, and that the incidence of adverse events in the riluzole group was about 32%. We performed a meta-analysis for the frequency of adverse events. The results showed that the group that received CHM plus a placebo for riluzole had fewer adverse events than the group that received riluzole plus a placebo for CHM (RR: 0.02; 95% CI: 0 to 0.14; *P* < 0.001; I^2^ = 0%; *n* = 2 RCTs; 136 participants) (23, 27). The CHM group had fewer adverse events than the riluzole group (RR:0.15; 95% CI: 0.07 to 0.3; *P* < 0.001; I^2^ = 0%; *n* = 3 RCTs; 166 participants) (24, 25, 28). Furthermore, there was no significant difference between CHM plus riluzole and riluzole (RR: 1; 95% CI: 0.36 to 2.75; *P* = 0.64; *n* = 1 RCT; 60 participants) (21). The results are shown in Supplementary Figure 8. The adverse events in the CHM group were mainly nausea (*n* = 3), abdominal distention (*n* = 2), and constipation (*n* = 2). The adverse events in the riluzole group were mainly nausea (*n* = 22), dizziness (*n* = 6), loss of appetite (*n* = 11), constipation (*n* = 3), diarrhea (*n* = 5), abdominal distention (*n* = 10), increased levels of transaminase (*n* = 49), fatigue (*n* = 11), and itchy skin (*n* = 4).

##### Other analysis

###### Subgroup analysis

The subgroup-analysis for ALSFRS or ALSFRS-r based on treatment duration and CHM preparation is shown in Table 3. The results of subgroup analyses suggested that there was a greater effect of CHM on improving functional ability in terms of ALSFRS or ALSFRS-r when the optimal therapeutic duration of DZSM seems to be 3 months or administered intravenously. Table 3 details the full statistical results of the subgroup analyses. For m-Norris, a subgroup analysis was not performed because of lack of sufficient number of studies.

**Table 3:** Subgroup analysis of ALSFRS or ALSFRS-r based on treatment duration and preparation of CHM.

**Subgroup**		**No. study**	**No. participants**	**Estimated effect (MD, 95% CI)**	***I^2^***	***P*** **value**	**Analysis model**
**CHM vs. placebo**						
Treatment duration	3 m	2	105	2.98 [0.77,5.19]	0%	< 0.01	Fixed model
	6 m	1	45	2.88 [-1.38,7.14]	-	0.18	Fixed model
	9 m	1	45	5.00 [-0.08,10.08]	-	0.05	Fixed model
Preparation of CHM	Decoction or granules	2	121	0.69 [0.42,0.97]	43%	< 0.01	Fixed model
	Injection	1	60	2.78 [0.15,5.41]	-	0.04	Fixed model
**CHM vs. riluzole**						
Treatment duration	3 m	2	108	2.68 [0.81,4.55]	0%	< 0.01	Fixed model
	6 m	1	42	3.80 [-1.41,9.01]	-	0.15	Fixed model
Preparation of CHM	Decoction	1	48	1.80 [-2.62,6.22]	-	0.42	Fixed model
	Injection	1	60	2.87 [0.81,4.93]	-	< 0.01	Fixed model

T, treatment group; CHM, Chinese herbal medicine; ALSFRS, ALS functional rating scales; ALSFRS-r, revised ALS functional rating scales; m, months; MD, mean difference.

###### Sensitivity analysis and publication bias

Because of the lack of a sufficient number of trials, we could not perform an additional analysis.

## Discussion

### Summary of findings and applicability

We comprehensively and systematically searched electronic databases to perform the updated meta-analysis to evaluate the efficacy and safety of CHM for ALS. Conventional pharmacotherapies may sometimes fail to cure ALS, such as riluzole or other antixodants with disadvantages of being expensive and having side effects and uncertain efficacy, whereas ALS is reported to be improved with alternative and complementary medicine based on traditional Chinese theory (4). Although there have been some systematic reviews or scoping reviews on CHM, no study evaluated the overall efficacy and safety of Chinese patent medicines, traditional herbal decoctions, or Chinese medicine compounds for ALS based on a meta-analysis.

In our study, we used the ALSFRS and m-Norris scales as the primary outcome to assess the efficacy of CHM for ALS function because these scales are the most representative tools to evaluate the physical function of daily life in ALS clinical trials, with tested reliability, sensitivity, and stability (30, 31). Our findings showed that CHM was superior to both the placebo and riluzole in terms of ALSFR and was superior to riluzole alone when used as an add-on therapy in terms of ALSFR after 3 months of treatment. Our subgroup analysis results suggest that 3 months of treatment can significantly improve the ALSFRS or ALSFRS-r scores, and that the different administrations of CHM may lead to different effects. These data imply that the course of treatment may affect the efficacy of functional change in patients with ALS as measured by ALSFRS or ALSFRS-r. Furthermore, we found that the effect was similar between the CHM group and the placebo or riluzole group with regard to improvements in m-Norris score after treatment regardless of the length of treatment. However, the change in m-Norris score from baseline to the end of more than 3 months of treatment was significantly different when compared between the CHM plus CM group and the CM alone group. This means that the determination of outcome measures may lead to different results.

The other findings in this review, which estimated the effect on secondary outcome, revealed that CHM had a better effect than the placebo and CM with regard to increasing the clinical effective rate and was superior to CM alone when used as an add-on therapy. In terms of TCM syndrome scores, CHM also had a better effect than CM although there was no obvious advantage of CHM with regard to improving fleshy atrophy symptoms and limb wilt symptoms in the TCM system. Extensive research is still needed to demonstrate the effectiveness of CHM in improving TCM syndrome scores. Only one RCT featuring a double blind and double dummy design reported improvement in FVC values (27). However, the data do not mean that CHM could improve pulmonary function in the clinic because we only used FVC as an evaluation measure. The existing evidence is insufficient to prove the efficacy of CHM in terms of improving pulmonary function. In addition, patients appear to be concerned about whether CHM can prolong survival, reduce mortality, or improve life quality. Our results showed that CHM did not significantly improve life quality and did not reduce endpoint events such as mortality and the need for tracheotomy. Therefore, we did not find any evidence to support the fact that CHM did provide a greater benefit with regard to prolonging survival or improving life quality. A more rigorous and multi-center study on a larger number of subjects is required to prove the efficacy in the future.

A previous investigation from China showed that the mean price of TCM decoctions and compounds range from 10.23 to 72.87 RMB/per day, and that riluzole costs 160 RMB/per day (7). The trial in our included studies also reported that CHM was cheaper than riluzole (28). Therefore, CHM could be a promising therapy for ALS because of its considerable efficacy and appreciable price. Moreover, this treatment option is safe as severe adverse events have not been reported. Moreover, there were fewer adverse events in the CHM group.

### Interpretation of different CHM potential mechanisms

The mechanisms underlying the effects of CHM prescriptions when dealing with ALS remain incompletely clear; some studies, however, provide evidence for the relationship between CHM intake and ALS prognosis, as well as the mechanisms underlying the effects.

Our studies demonstrated that the most frequent formulae were Jia Wei Si Jun Zi decoction. Zhu observed the effect of supplementary Sijunzi decoction on SOD1 transgenic ALS mice and explored the mechanisms involved; the results showed that the Sijunzi decoction may reduce the abnormal aggregation of abnormally phosphorylated neurofilaments around the nuclei probably by inhibiting the abnormal phosphorylation of neurofilament, thus maintaining the integrity of the cytoskeleton, alleviating axonal atrophy, improving axonal transport, delaying neuronal degeneration, and exerting neuroprotective effects (9).

ALS is a form of a wilt disease in traditional medicine (32). TCM physicians believe that the pathogenesis of a wilt disease is mostly related to deficiencies in the spleen, kidneys, lungs, and liver, and consider that the therapeutic principles of invigorating the spleen, soothing the liver, and tonifying the kidneys and lungs are beneficial for ALS from a syndrome differentiation point of view; thus, therapeutic prescriptions such as Jianpi Bushen, Jianpi Yifei, and Bushen Jianpi Shugan prescriptions are used extensively. Zhu et al. showed that Jianpi Bushen prescription could improve the activity ability as well as balance in a ADAR2-knockout ALS mouse; this may be associated with the delayed degeneration and loss of anterior horn neurons as well as the inhibition of ADAR2 immunoreaction generated by CHM (33). Pan et al. found that Jianpi Yifei prescription may play a neuroprotective role by inhibiting the activation of microglia and by downregulating the expression of the p38 MAPK protein and inflammatory factors by mediating the p38 MAPK pathway in a mouse model of ALS (34). These results demonstrate the potential of a CHM prescription as a supplementary treatment for ALS.

A previous study analyzed a CHM prescription for a wilt disease and ALS by data mining and found that the commonly used herbs were *dangshen, huangqi, fuling, baizhu, dihuang*, and *danggui* (35); the results were consistent with our present findings. The TCM theory suggests that *dihuang* can tonify the kidney, nourish *yin*, and fill lean pulps, that *dangshen* can replenish the spleen and lung *qi*, that *huangqi, fuling*, and *baizhu* can tonify *qi* and strengthen the spleen, and that *danggui* can tonify blood circulation and relieve pain. Evidence from modern medicine shows that the pathogenesis of ALS mainly involves excitotoxicity, oxidative stress, mitochondrial abnormality, and immune inflammatory response (36). Patients with ALS have elevated levels of glutamate (Glu) in the cerebrospinal fluid and elevated levels of Glu release were found in the spinal cord of ALS transgenic mice (37), thus aggravating excitotoxicity. It has been shown that *dihuang* extract exerts cytoprotective effects on Glu-induced PC12 cytotoxic injury *via* pathways related to energy metabolism (38). *Dangshen, fuling*, and *baizhu* may benefit ALS by antioxidation (39–42). On the other hand, the combination of *huangqi* and *danggui* may be beneficial for ALS by regulating the gene expression of T cells and cytokines, thus playing a role in immune regulation (43, 44).

### Strengths of this study compared to those published previously

A previous systematic review and meta-analysis on tonic class prescriptions for ALS was published in 2016 and showed that CHM was superior to conventional therapy in terms of improving clinical effects and m-Norris scale score (45). However, our present study is very different. First, the previous studies did not register the associated protocol in advance on PROSPERO or other platforms and used the Jadad scale to assess the quality of studies. In our study, we registered our review protocol on PROSPERO (Reference: CRD42022323047) to avoid selection bias and used the Cochrane risk and bias tool to evaluate the quality of the included studies. Second, we performed our systematic and comprehensive literature search from inception to March 2022 to gather more up-to-date trials from six databases. We only included Chinese herbal compounds or decoctions and excluded other forms of Chinese medicines such as acupuncture and massage. Third, we assessed different outcomes from different perspectives especially patient-centered outcomes such as mortality and quality of life.

### Limitations and implications for research

There are some limitations associated with our meta-analysis that need to be considered, i.e., the included trials were conducted in China. In the future, we must incorporate more RCT results from other countries or different races. We also restricted the retrieval language to both Chinese and English; this may have led to a potential publication bias. ALS is also a progressive disease; we did not collect detailed characteristics of the disease or grade the severity of ALS. Therefore, we were unable to evaluate the efficacy of CHM for different grades of ALS. Furthermore, the outcomes were mainly subjective, and the blinding of outcome assessors was mostly unclear; this may have affected the credibility of the efficacy data. Further studies need to evaluate hard endpoints such as survival and mortality or consider objective indices such as electromyogram results. Future research should exhibit more conformity in terms of outcome measures. Furthermore, the designs of the included studies were relatively poor; future studies should involve randomization and triple blinding and recruit a larger number of participants to conduct a more rigorous and methodological study. Personnel should perform follow-up studies to demonstrate the long-term efficacy of CHM. We were unable to identify a specific important ingredient. However, Jia Wei Si Jun Zi decoctions, Ji Wei Ling preparations, and Fu Yuan Sheng Ji granules had relatively more studies in our review, and subjects numbers should be priorities for further research. It is also hoped that future research will continue to explore the mechanisms and efficacy of specific drugs or active ingredients.

## Conclusion

Our study suggests that short-term adjunct use of CHM could improve ALSFRS score and exert beneficial clinical effects with a good safety profile when compared with a placebo or riluzole alone. However, future research or clinical studies should focus on the long-term efficacy of patient-oriented outcomes.

## Data availability statement

The original contributions presented in the study are included in the article/Supplementary material, further inquiries can be directed to the corresponding author/s.

## Author contributions

LG and QC conceived and designed the study. YL and SH drafted the manuscript, screened the studies, and extracted the data. YL performed the meta-analysis. DLiu, SY, and DLi interpreted the results and revised the manuscript. All authors have read and agreed to the published version of the manuscript.

## Funding

This study was funded and supported by the Yunnan Provincial Science and Technology Department-Applied Basic Research Joint Special Funds of Chinese Medicine (Youth Project: 202101AZ070001-127) and by Kunming Health Science and Technology Talents Training Project (Number: 2019-SW-province-20).

## Conflict of interest

The authors declare that the research was conducted in the absence of any commercial or financial relationships that could be construed as a potential conflict of interest.

## Publisher's note

All claims expressed in this article are solely those of the authors and do not necessarily represent those of their affiliated organizations, or those of the publisher, the editors and the reviewers. Any product that may be evaluated in this article, or claim that may be made by its manufacturer, is not guaranteed or endorsed by the publisher.

## Appendix

### Search strategy

#1 “amyotrophic lateral sclerosis”[MeSH Terms]

#2 “randomized controlled trial”[tw] OR “controlled clinical trial”[tw] OR “randomized”[tw] OR “placebo”[tw] OR “randomly”[tw]

#3 (((((((“Medicine, Chinese Traditional”[Mesh]) OR “Drugs, Chinese Herbal”[Mesh]) OR “Medicine, Traditional” [Mesh]) OR “Plants, Medicinal” [Mesh]) OR “Phytotherapy” [Mesh]) OR “Pharmacognosy” [Mesh]) OR “Plant Extracts” [Mesh]) OR “Ethnopharmacology” [Mesh]

#4 (traditional [tw] or Chinese [tw] or “traditional Chinese” [tw] or folk [tw] or therapy [tw] or preparation [tw]) and (drug? [tw] or herbal [tw] or plant? [tw] or extract? [tw]) or indigenous [tw] or Kampo [tw] or Kanpo [tw]) and (medici*[tw] or remedies[tw] or herb? [tw] or pharmaceutical [tw] or healing [tw])

#5 #3 OR #4

#6 #1 AND #2 AND #5
