# Supplementary material for: The efficacy and safety of Chinese herbal medicine as an add-on therapy for amyotrophic lateral sclerosis: An updated systematic review and meta-analysis of randomized controlled trials
Source: Front Neurol. 2022 Oct 6;13:988034. doi: 10.3389/fneur.2022.988034 (PMC9583903; doi:10.3389/fneur.2022.988034)
Supplement: Supplementary file 1 [file Data_Sheet_1.PDF]

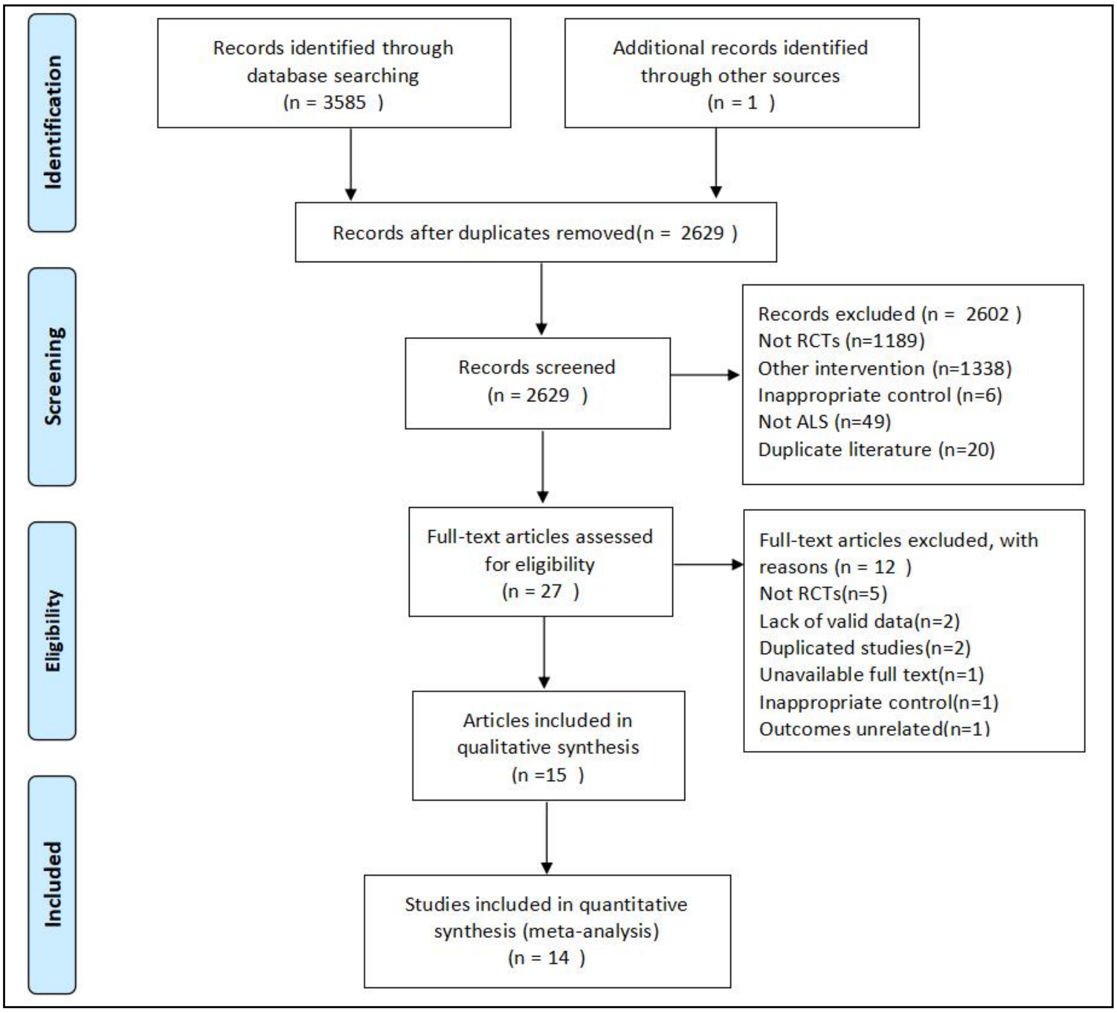

Supplementary Figure 1. Flowchart of studies screening

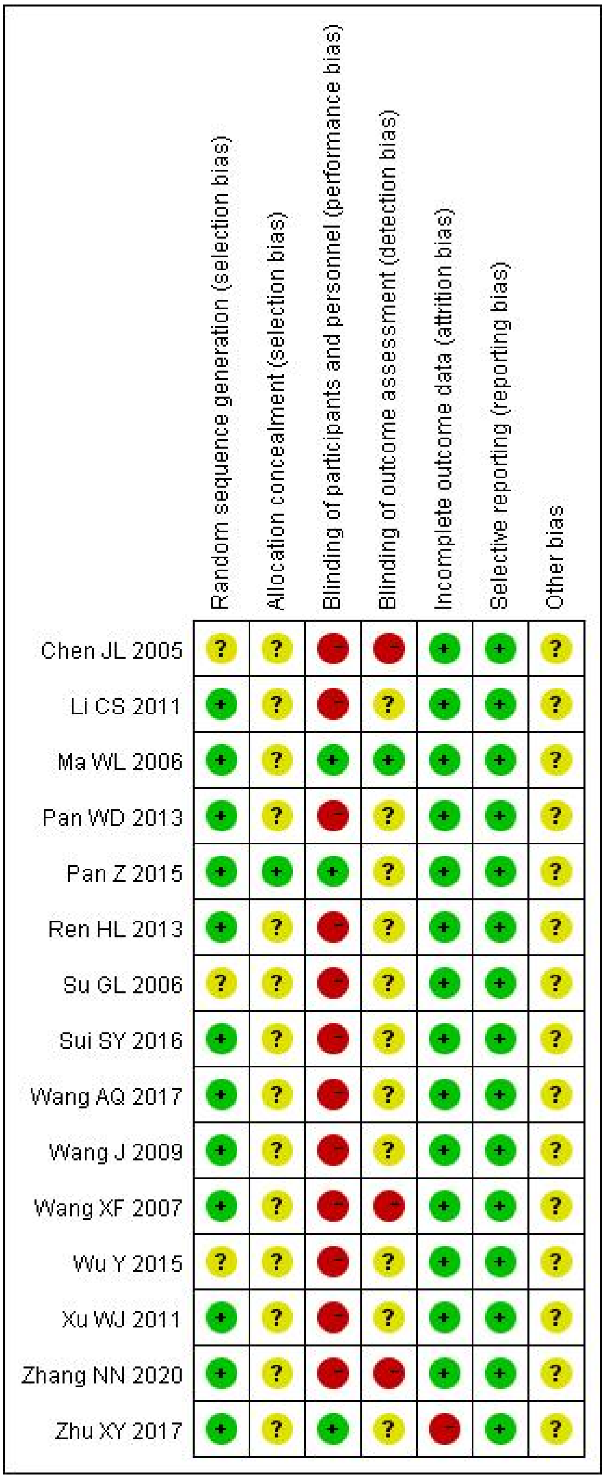

Supplementary Figure 2 The risk of bias summary of all included studies

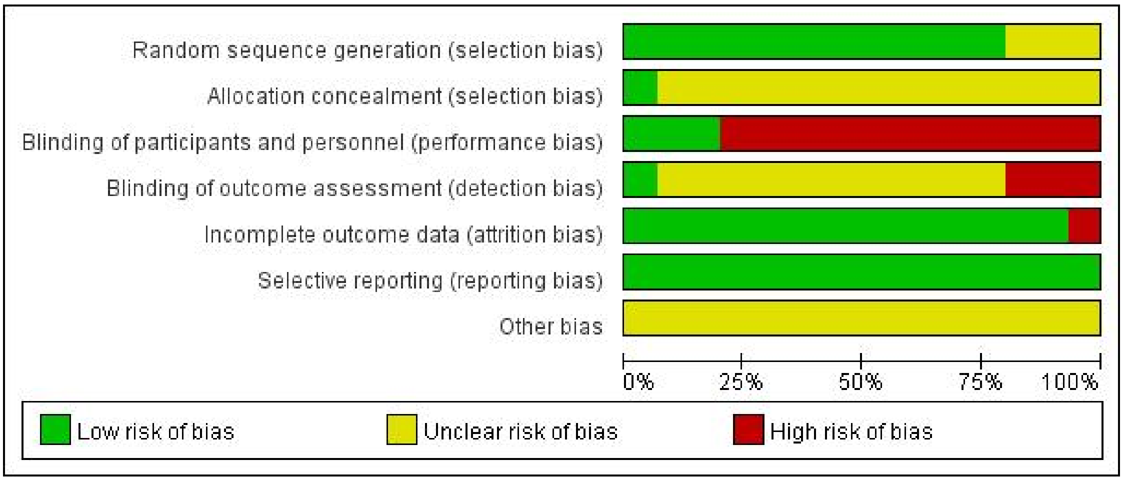

Supplementary Figure 3 The risk of bias graph of all included studies

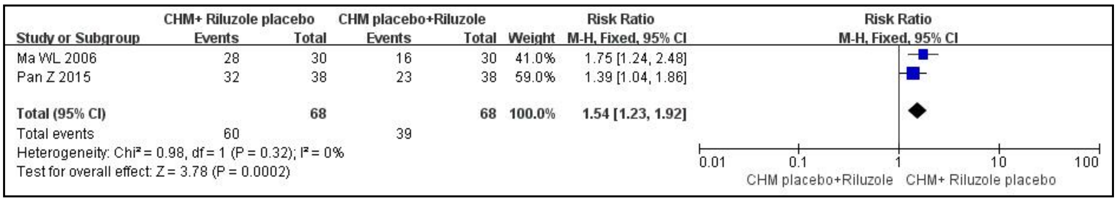

Supplementary Figure 4 Forest plot of clinical effect for ALS compared CHM plus riluzole placebo with CHM placebo plus riluzole. CHM: Chinese herbal medicine; ALS:Amyotrophic lateral sclerosis;

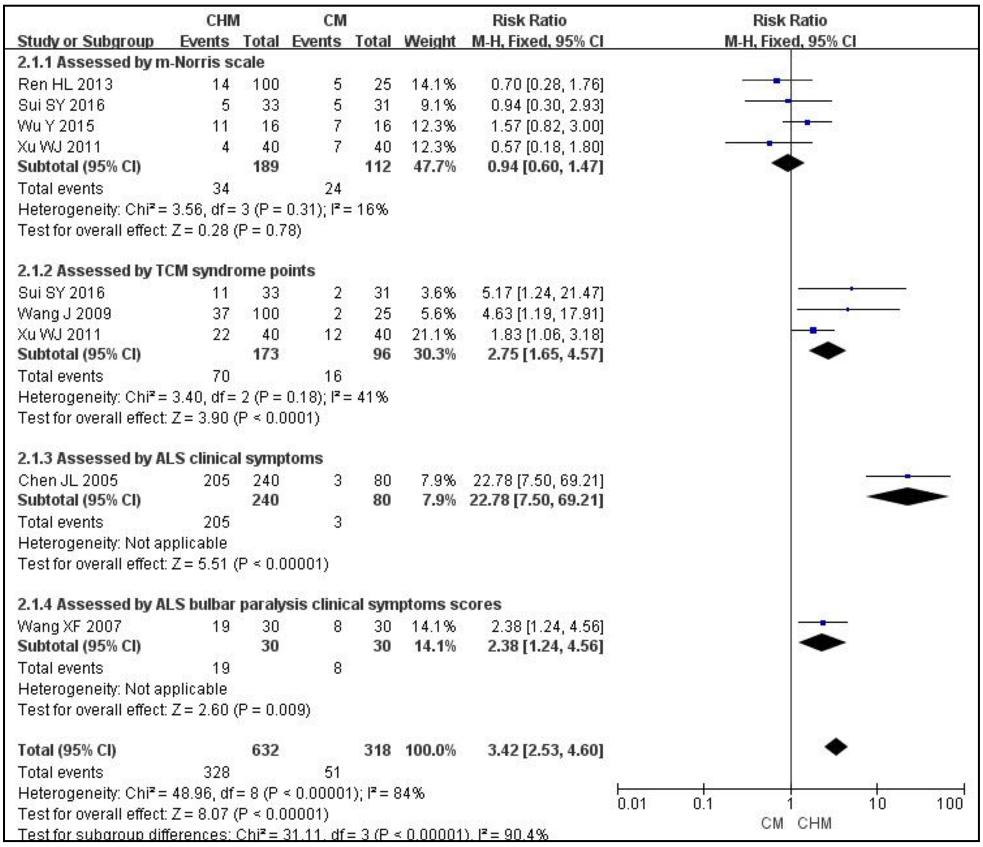

Supplementary Figure 5 Forest plot of clinical effect for ALS compared CHM with CM. CHM: Chinese herbal medicine; ALS:Amyotrophic lateral sclerosis; CM:Conventional medicine

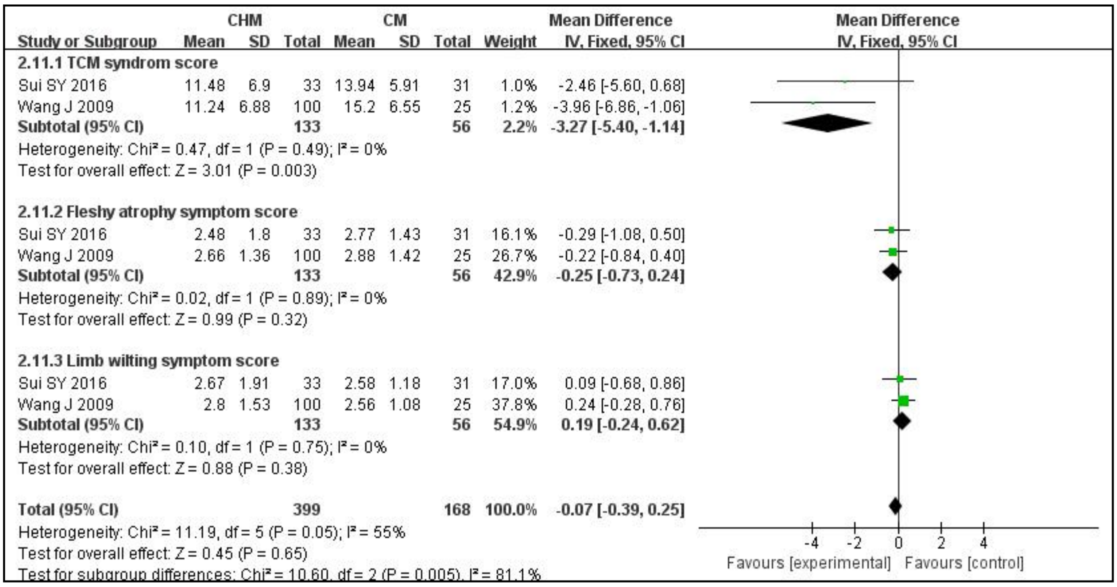

Supplementary Figure 6 Forest plot of TCM syndrome score for ALS compared CHM with CM. CHM: Chinese herbal medicine; ALS:Amyotrophic lateral sclerosis; CM:Conventional medicine; TCM: Traditional Chinese Medicine

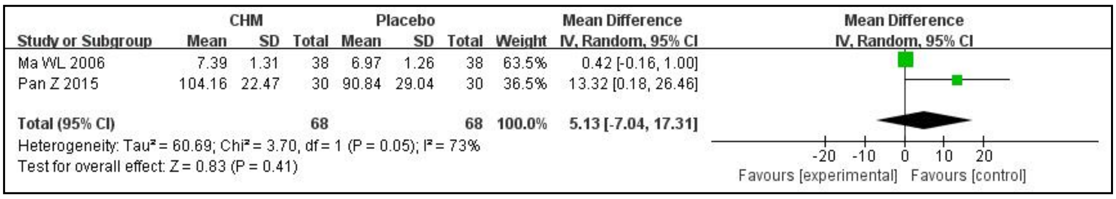

Supplementary Figure 7 Forest plot of life quality for ALS compared CHM with controls. CHM: Chinese herbal medicine; ALS:Amyotrophic lateral sclerosis

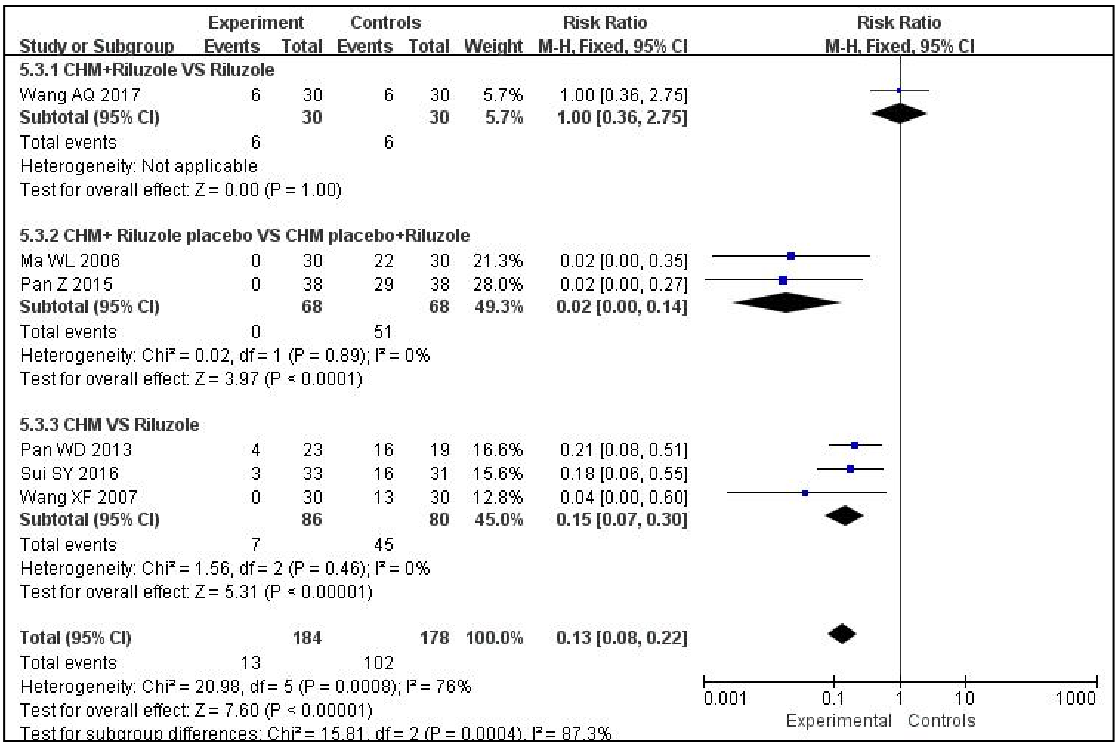

Supplementary Figure 8 Forest plot of adverse events for ALS compared experimental with controls. CHM: Chinese herbal medicine; ALS:Amyotrophic lateral sclerosis; CM: Conventional medicine
